# Impact of Pre-Analytical Variables on Cancer Targeted Gene Sequencing Efficiency

**DOI:** 10.1371/journal.pone.0143092

**Published:** 2015-11-25

**Authors:** Luiz H. Araujo, Cynthia Timmers, Konstantin Shilo, Weiqiang Zhao, Jianying Zhang, Lianbo Yu, Thanemozhi G. Natarajan, Clinton J. Miller, Ayse Selen Yilmaz, Tom Liu, Joseph Amann, José Roberto Lapa e Silva, Carlos Gil Ferreira, David P. Carbone

**Affiliations:** 1 James Thoracic Center, The Ohio State University Comprehensive Cancer Center, Columbus, Ohio, 43210, United States of America; 2 Department of Biomedical Informatics, The Ohio State University, Columbus, Ohio, 43210, United States of America; 3 GenomOncology, Cleveland, Ohio, 44145, United States of America; 4 Biomedical Informatics Shared Resource, The Ohio State University Comprehensive Cancer Center, Columbus, Ohio, 43210, United States of America; 5 Federal University of Rio de Janeiro, Rio de Janeiro, Brazil; 6 Brazilian National Cancer Institute, Rio de Janeiro, Brazil; Virginia Commonwealth University, UNITED STATES

## Abstract

Tumor specimens are often preserved as formalin-fixed paraffin-embedded (FFPE) tissue blocks, the most common clinical source for DNA sequencing. Herein, we evaluated the effect of pre-sequencing parameters to guide proper sample selection for targeted gene sequencing. Data from 113 FFPE lung tumor specimens were collected, and targeted gene sequencing was performed. Libraries were constructed using custom probes and were paired-end sequenced on a next generation sequencing platform. A PCR-based quality control (QC) assay was utilized to determine DNA quality, and a ratio was generated in comparison to control DNA. We observed that FFPE storage time, PCR/QC ratio, and DNA input in the library preparation were significantly correlated to most parameters of sequencing efficiency including depth of coverage, alignment rate, insert size, and read quality. A combined score using the three parameters was generated and proved highly accurate to predict sequencing metrics. We also showed wide read count variability within the genome, with worse coverage in regions of low GC content like in *KRAS*. Sample quality and GC content had independent effects on sequencing depth, and the worst results were observed in regions of low GC content in samples with poor quality. Our data confirm that FFPE samples are a reliable source for targeted gene sequencing in cancer, provided adequate sample quality controls are exercised. Tissue quality should be routinely assessed for pre-analytical factors, and sequencing depth may be limited in genomic regions of low GC content if suboptimal samples are utilized.

## Introduction

In the past decade, a better understanding of cancer biology and the identification of somatic mutations in cancer have led to a new era in personalized oncology.[1] Landmark examples included the discovery of mutations in the proto-oncogenes c-*KIT* in gastrointestinal stromal tumors (GIST),[2] epidermal growth factor receptor (*EGFR*) in lung adenocarcinomas,[3] and v-Raf murine sarcoma viral oncogene homolog B1 (*BRAF*) in melanomas.[4] Tumors harboring these mutations demonstrate outstanding sensitivity to specific kinase inhibitors directed against the respective activated pathways.

These oncogenic mutations are often defined as cancer drivers since they offer a selective advantage to a cell clone, necessary for tumor initiation and maintenance.[5] In the clinic, they may serve as fingerprints that help clinicians to subtype cancers that, otherwise, present similar histological patterns.[6–9] While mutational profiling has become a useful tool to better tailor targeted therapies, new challenges have arisen including the frequent need to obtain optimal tumor specimens for extra genetic testing.[10–12] Moreover, multiple tests may be recommended in a clinical setting where a plethora of candidate driver mutations need to be assessed. In non-small cell lung cancer (NSCLC), alterations in at least 10 proto-oncogenes have been suggested as potentially “druggable”, with mutation frequencies varying from 1% to 25% for *MAP2K1* and *KRAS*, respectively, according to the population studied.[13] The best algorithm for testing, including sequential versus multiplex assessment of such alterations is still a matter of debate.

Although Sanger sequencing has been traditionally used for the detection of recurrent point mutations in cancer, newer technologies have enabled a more comprehensive analysis of genetic perturbations. In this scenario, the next generation sequencing (NGS) platforms—also known as massively parallel sequencing—offer a wide range of opportunities to characterize the cancer genome.[14–16] For instance, the availability of hybridization-capture techniques provides a high-throughput and cost-effective strategy to assess hundreds of genes simultaneously.[16–19] As a brief methodological review, genomic DNA (gDNA) is purified from tumor samples and sheared by either sonication or restriction enzymes into millions of small fragments (tens or hundreds nucleotides long). These fragments are then hybridized to a customized probe set that contains baits specific for the genes of interest, and amplified to generate the sequencing library. A unique barcode is ligated to each library—corresponding to each sample—which enables multiple samples to be pooled together for sequencing. Several commercial DNA capture technologies are currently available, and many institutions have designed and implemented customized targeted panels to genotype cancer samples.[10, 20, 21]

Clinical tumor specimens are often preserved as formalin-fixed paraffin-embedded (FFPE) tissue blocks in biorepositories, and this is the most readily available source for obtaining gDNA both in clinical and research settings.[22–24] However, several steps in the FFPE processing are known to cause DNA damage, which directly affect DNA quality and adequacy for sequencing. For instance, formalin fixation may result in various types of crosslinks between two amino acids, two nucleic acids, or between an amino acid and a nucleic acid base.[25–27] These chemical modifications may confound molecular testing through inhibition of enzymatic manipulation of DNA. Formalin fixation may also cause nucleotide oxidation and deamination, the latter being related to the development of artifactual nucleotide transitions (mostly C>T in CpG dinucleotides) among samples stored as FFPE.[23, 28] Lastly, methylene crosslinks caused by formalin may result in DNA fragmentation, which limits the DNA length for sequencing. In addition to formalin fixation, tissue preparation, paraffin embedding, and archival storage *per se* may all ultimately play a role in samples quality.[29] Moreover, FFPE blocks are often obtained from small biopsies, and low tissue quantity may constitute an additional limitation for sequencing. In order to evaluate the quality of gDNA extracted from FFPE samples, PCR-based quality control (QC) assays have been recommended.[30–33] Other variables likely to affect final sequencing results include the amount of DNA used as input for the library preparation, the sequencing depth, and the targeted region of interest (GC content and sequence homology).

Herein, we evaluated the individual and combined impact of pre-sequencing parameters on targeted gene sequencing efficiency. To this end, we utilized a fully annotated sample set characterized by knowledge of a wide range of pre-analytical variables, which was genotyped for a customized gene panel using a commercially available targeted gene sequencing approach—the Agilent Haloplex Target Enrichment System (Agilent Technologies). This platform differs from other hybridization-capture techniques in that a pool of restriction enzymes is used to digest the sample DNA (as opposed to sonication), and the probes are designed with homology only to the ends of targeted DNA restriction fragments.[34] Subsequently, universal primers are used to amplify the captured regions and will generate a high frequency of similar reads, resembling the results found in amplicon-based platforms (S1 Fig). For this reason, some sequencing metrics like duplication rate and unique reads quantification are not applicable to this technology. We also verified the read depth variability within the genome, disclosed problematic regions based on GC content, and determined the impact of these parameters on variant calling. These data could be very informative to guide the clinical and research community in the adequate selection of clinical samples for targeted gene sequencing, and in the proper interpretation of sequencing results as a function of sample quality and sequencing uniformity.

## Materials and Methods

### Clinical samples

The studied dataset comprised 113 lung tumor specimens resected from patients at the James Cancer Hospital / The Ohio State University (OSU, Columbus, OH) between 1988 and 2011. All the samples were archived as FFPE tumor blocks, and were selected based on tissue availability. One hundred and ten samples were primary NSCLC (60 adenocarcinomas, 31 squamous cell carcinomas, 10 adenosquamous, and 9 other histological subtypes), while 3 samples were head and neck cancers (all squamous cell carcinomas) metastasizing to the lungs (Table A in S1 File). Each sample was assigned a unique, unidentifiable code, and date of surgery was reviewed and annotated to estimate the tumor block storage time. The Institutional Review Board approved this project, and waived the need for consenting.

### Tissue processing

Resected samples with representative tumor tissue were selected for NGS testing. To increase tumor content, a pathologist (K.S.) marked an H&E stained slide to delineate tumor-containing regions, and these areas were macrodissected by manual scraping the marked areas from serial unstained FFPE sections. Tumor cellularity was determined by visual inspection of the number of tumor nuclei compared to stromal background in the areas marked for macrodissection, and most samples (88%) were classified as containing either high or moderate tumor cellularity (Table B in S1 File and S2 Fig). gDNA was extracted from FFPE samples using the Maxwell^®^ 16 FFPE Plus LEV DNA Purification kit (Promega). Two to ten slides containing 10μm thick sections were scraped into a microtube and incubated overnight at 70°C with proteinase K solution and incubation buffer. Subsequently, each sample was treated with lysis buffer, transferred to loading cartridges and run in the automated instrument. Local testing showed that this protocol yielded similar amounts of DNA in comparison to manual systems (data not shown). gDNA quantification was performed using the Quant-iT^™^ High-Sensitivity DNA Assay Kit (Life Technologies^™^).

### DNA quality assessment

In order to determine overall quality of the gDNA, a PCR-based QC assay was applied, and used as a guide to recommend the amount of DNA input in the library preparation, as recommended by the manufacturer.[35] Briefly, 10 ng of each DNA sample was amplified with 2 independent primer pairs in order to generate amplicons of incremental sizes: 105 base pairs (bp), and 236 bp. As a non-degraded positive control, we used gDNA extracted from a NSCLC cell line (A549). After PCR, products were evaluated for yield and level of fragmentation using the Agilent 2200 TapeStation (Agilent Technologies). The QC ratio was calculated dividing the bands quantification for each sample by the respective band in the positive control, and then averaging each band ratio. A QC ratio above 0.20 indicates favorable quality, while ratios below 0.20 suggests moderate or poor quality.[35]

### Hybridization-capture and sequencing

A custom panel was designed using the internet-based Sure Design software (Agilent Technologies) to cover the coding regions of 81 selected genes relevant to NSCLC (Table C in S1 File). The total panel covered 920,980 base pairs, and included 44,234 amplicons. Libraries were constructed and indexed using the Agilent Haloplex Target Enrichment System (Agilent Technologies). The indexed libraries were pooled at equimolar amounts and paired-end sequenced (2 x 100 base pairs) to 1,000X average coverage on an Illumina HiSeq 2500.

### Data processing

Sequencing reads were aligned to the human genome (hg19 assembly) and bam files were generated using the SureCall software (Agilent Technologies). Variant calling was performed using the Genome Analysis Tool Kit (GATK) Unified Genotyper. Variant annotation was carried out on GenomOncology’s GenomAnalytics platform (GenomOncology, Cleveland, OH), and the Integrative Genomic Viewer (IGV, Broad Institute) was used to confirm true positives. Sequencing performance was assessed by measuring the number of reads, mapped reads, target base coverage, and read quality using Picard (Broad Institute), SAMtools and BEDTools.[36, 37] The depth of coverage in genomic regions was verified with the Depth Of Coverage tool (GATK),[38] and in specific genomic position (hotspots) using IGV.

### Statistical methods

Three pre-analytical variables were used to predict final sequencing depth—FFPE storage time, PCR/QC ratio, and DNA input. FFPE storage time in paraffin block was calculated as the interval (in years) from the date of surgery to date of tumor processing (DNA extraction) for sequencing. To test the impact of these variables on the overall sequencing efficiency, we used several parameters like depth of coverage, alignment rate, off-target rate, base quality, among others. The pairwise correlation between pre-analytical variables (storage time, PCR/QC ratio and DNA input) and sequencing performance parameters was evaluated by Pearson’s method. Subsequently, we created a training dataset comprising genomic alterations located in genes with the least coverage variability. Ten genes were filtered in: *ALK*, *BCL11A*, *REL*, *VGLL4*, *RAF1*, *FBLN2*, *RET*, *FGFR2*, *MAP2K1*, *U2AF2*. We then excluded genomic regions with overall poor coverage (less than 100 average reads) or with high depth variability (with standard deviation in the upper quartiles of variance) within these genes. These criteria led to a training dataset of 33 genomic regions, which was used to compare the pre-sequencing variables. Multivariable linear regression was performed for the correlation between the median reads and the three pre-analytical variables and for the potential multicollinearity among the three factors. This analysis indicated whether each covariate included in the model was still significantly correlated to sequencing performance after adjusting for other covariates. An equation based on the stepwise model selection procedure of all three individual factors and 2-way interaction terms was built to generate the combined score or the predictive sequencing performance. The final model selected the three individual pre-sequencing factors. The formula is presented here: *Combined score = 202*.*95–7*.86 * Storage time + 249.95 * PCR ratio + 0.08 * DNA input. In order to evaluate coverage variability within the genome (in GC content analysis), overall coverage was quantile-normalized and then stratified according to the GC content ratio. To compare the effect of GC content and tissue quality altogether, samples were stratified according to baseline quality (defined by the pre-sequencing combined score quartiles), and the overall depth of coverage was quantile-normalized within each group. P-value of ≤ 0.05 was considered statistically significant. Statistical analyses were performed using R version 3.0.1, SAS 9.3, and IBM SPSS version 22.0.

## Results

### Sample and sequencing parameters

We observed a large variation in the sample and pre-sequencing QC parameter across the selected samples (Table 1), including a range in FFPE storage time of 0.32 years to 24.22 years. The PCR-based QC assay indicated a median ratio of 0.19 (range 0.03–0.58), suggesting that the gDNA had a favorable quality in approximately half of the samples, while the other half had lower quality. The amount of gDNA used as input in the library preparation varied from 77 ng to 2,337 ng, with a median of 899 ng.

**Table 1:** Pre-analytical parameters and sequencing metrics.

Feature	Median (range)
**Pre-analytical parameters**	
FFPE storage time (years)	9.17 (0.32–24.22)
PCR/QC ratio	0.19 (0.03–0.58)
DNA input (ng)	899 (77–2,337)
**Sequencing metrics**	
Sequence reads and coverage	5,041,132 (1,464,386–7,738,458)
Reads mapped to genome	4,918,758 (1,148,705–7,597,557)
Bases mapped to genome	390,673,221 (86,893,349–608,314,077)
On-target bases mapped to genome	277,117,362 (63,725,143)
Alignment rate (%)	98.1 (78.4–98.9)
Target coverage	881x (204–1,373)
Fraction of bases covered ≥ 20x (%)	95.4 (78.9–98.8)
Fraction of bases covered ≥ 50x (%)	90.8 (66.7–97.7)
Fraction of bases covered ≥ 100x (%)	84.6 (52.1–95.1)
Insert size (in base pairs)	89.4 (73.5–120.7)
Mean base quality score (Phred score)	35.1 (32.4–35.7)
Mean quality score last 20 bases	31.6 (25.0–32.6)
Mean quality score last 10 bases	31.2 (23.8–32.2)
Fraction of bases ≥ Q30 (%)	98.5 (74.5–100)

Abbreviations: FFPE, formalin-fixed paraffin-embedded tissue blocks; PCR/QC, PCR-based quality control; Q30, Phred scale quality of 30.

The median number of paired end reads and mapped reads per sample were 5.0 millions (range 1.4–7.7) and 4.9 millions (range 1.1–7.6), respectively, and 98.1% (range 78.4–98.9) of reads mapped to the target region. The median actual target coverage was 881X (range 204–1,373), with the median percentages of target reads covered at least 20-fold (20x), 50-fold (50x), and 100-fold (100x) being 95.4% (range 78.9–98.8), 90.8% (range 66.7–97.7), and 84.6% (range 52.1–95.1), respectively. The median insert size was 89.4 base pairs (range 73.5–120.7), and 98.5% of base calls had a Phred quality score of at least 30. These parameters are summarized in Table 1 and S3 Fig.

### Correlation between pre-analytical variables and sequencing efficiency

**Fig 1:**
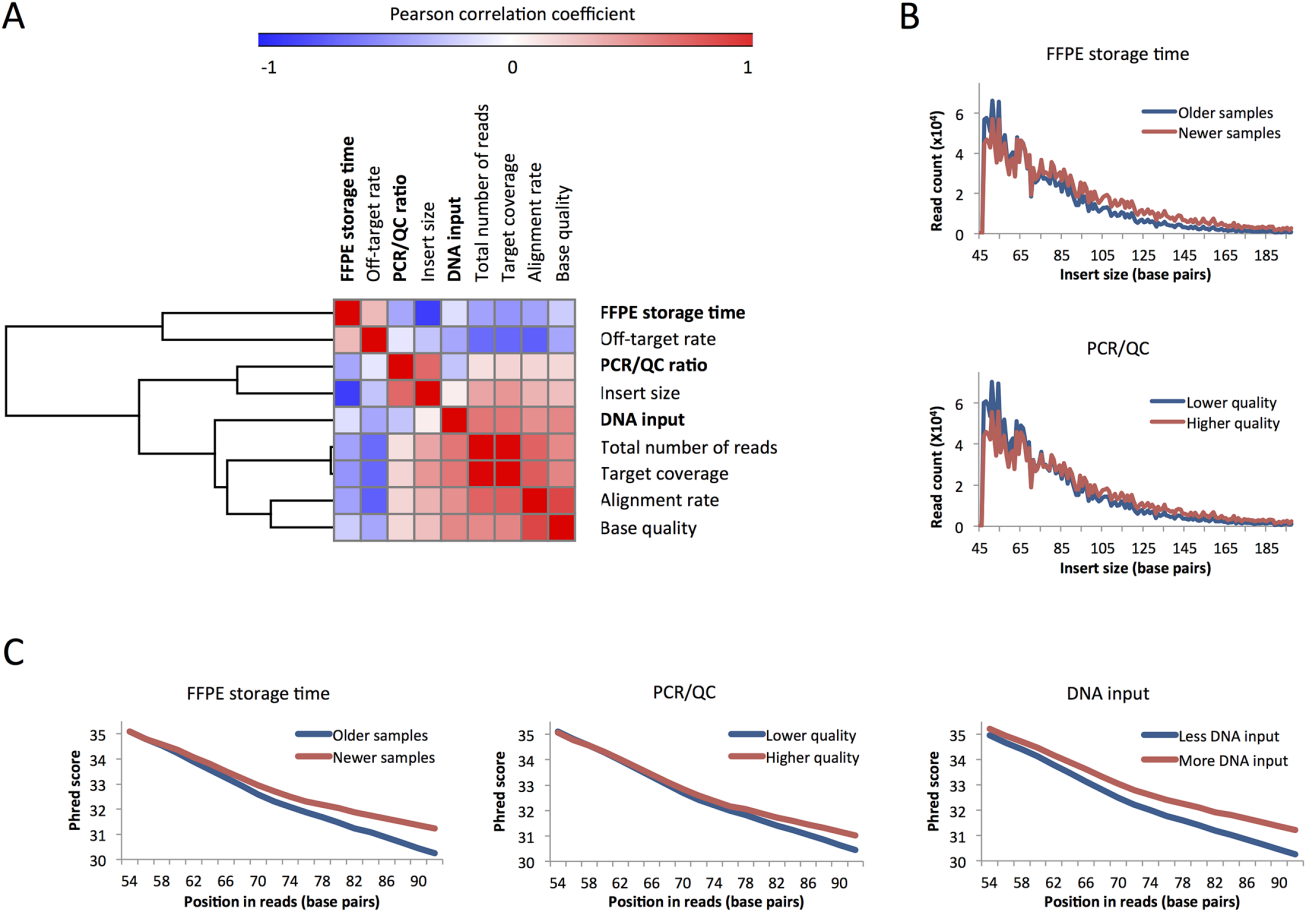
Three pre-analytical variables (FFPE storage time, PCR/QC ratio, and DNA input in the library preparation) were significantly correlated to most post-sequencing parameters (A). The pre-analytical variables were classified as below or above median values to illustrate the impact on insert size (B) and on read quality/Phred score (C). Abbreviations: FFPE, formalin-fixed paraffin-embedded tissue blocks; PCR/QC, PCR-based quality control.

The pre-analytical variables (FFPE storage time, PCR/QC ratio, and DNA input) were significantly correlated to most parameters of sequencing efficiency (Fig 1 and Table D in S1 File). FFPE storage time was negatively correlated to total number of reads (*r* = -0.356), mean target coverage (*r* = -0.405), alignment rate (*r* = -0.354), insert size (*r* = -0.764, p<0.01 in all cases), and mean base quality (*r* = -0.188, p = 0.046), and positively correlated to off-target rate (*r* = 0.285, p<0.01), compatible with better results if more recent samples are selected. The QC ratio was correlated to insert size (*r* = 0.601, p<0.01), and insignificantly correlated to target coverage (*r* = 0.183, p = 0.058), alignment rate (*r* = 0.169, p = 0.08), and base quality (*r* = 0.162, p = 0.094). DNA input was correlated to total number of reads (*r* = 0.548), mean target coverage (*r* = 0.549), alignment rate (*r* = 0.449), mean base quality (r = 0.477), and off-target rate (*r* = -0.336, p<0.01 in all cases), but not to insert size (*r* = 0.081, p = 0.395). These data suggest that better QC ratio and higher DNA input in the library preparation may predict greater sequencing efficiency.

### The combined effect of pre-analytical variables

**Fig 2:**
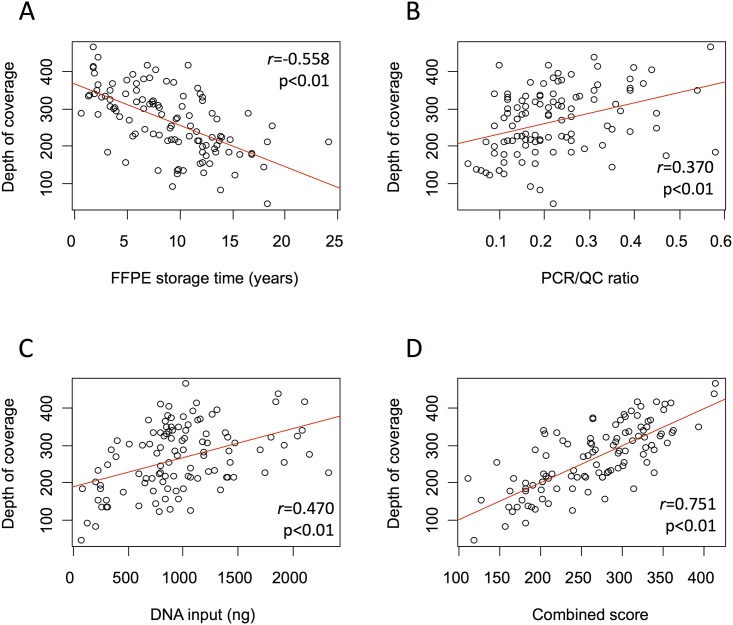
FFPE storage time (A), PCR/QC ratio (B), and DNA input (C) were correlated to sequencing depth of coverage. A combined score (D) was constructed based on these three parameters, and was highly correlated to sequencing depth. Abbreviations: FFPE, formalin-fixed paraffin-embedded tissue blocks; PCR/QC, PCR-based quality control.

**Fig 3:**
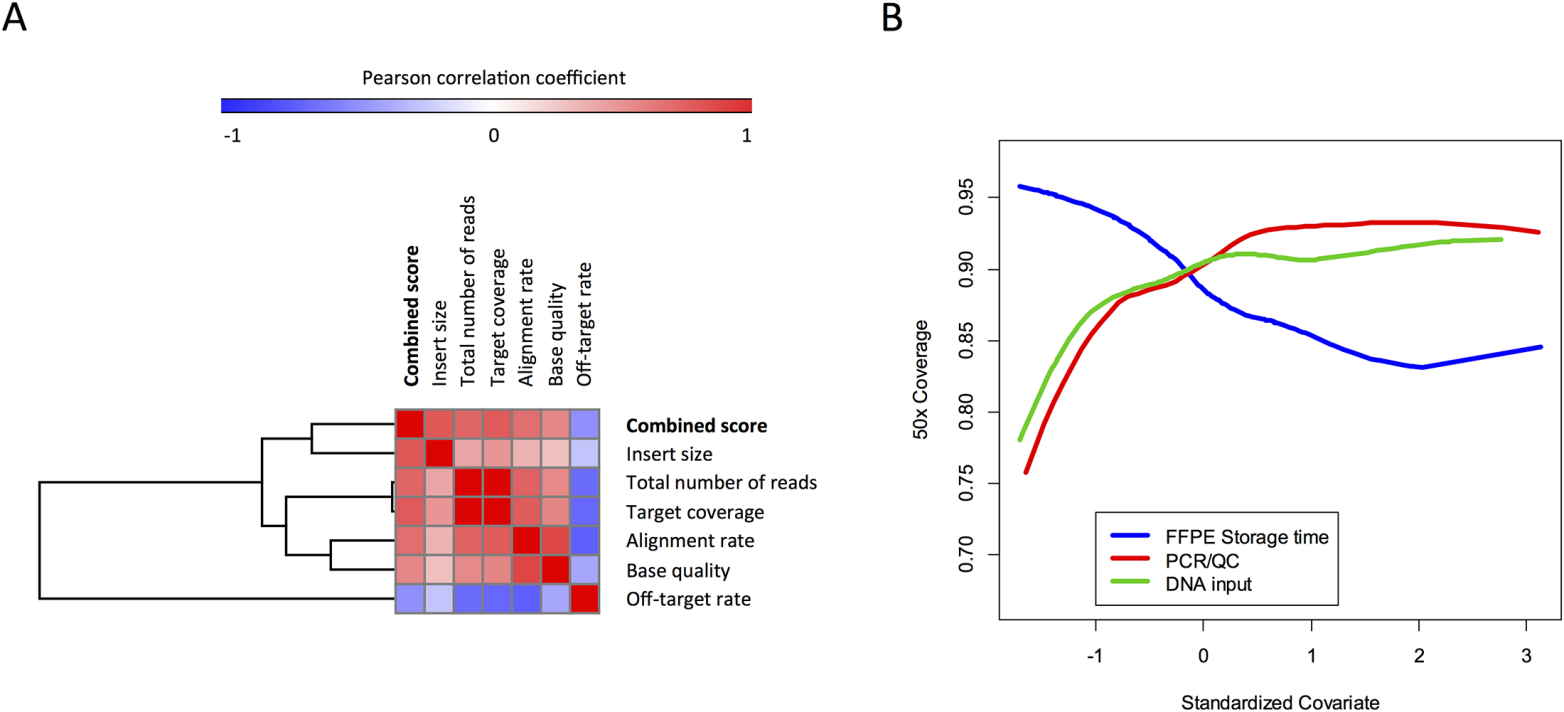
The combined score was strongly correlated to post-sequencing parameters (A). Correlation to the 50x coverage was used to define pre-analytical thresholds that could predict sequencing efficiency, and are illustrated by smoothing curves (B).

The combined effect of pre-analytical variables was assessed in a training dataset of 33 genomic regions, with median depth of coverage of 267 (range 43–464). FFPE storage time was negatively correlated to the depth of coverage (*r* = -0.558, p<0.01; Fig 2A), while QC ratio and DNA input were positively correlated (*r* = 0.37 and 0.47, respectively; p<0.01 in both; Fig 2B and 2C). Using a multivariate analysis, we showed that each of these variables was still significantly correlated to sequencing performance (Table E in S1 File). In order to generate a unique score that predicts sample quality in this cohort, we merged all the three variables into a combined score, as described in methods. As expected, the combined score was highly correlated to the depth of coverage in the training dataset (*r* = 0.751; p<0.01; Fig 2D). To confirm its accuracy, we compared it to the mean target coverage in all studied genomic regions (independently of bias caused by copy number alterations) and to the read depth in bases harboring frequent germline or somatic single nucleotide variations (SNV), located in genes not used in the training dataset. There was strong positive correlation between the combined score and the depth of coverage in all these instances. In addition, we demonstrated strong correlation between the combined score and the 20x, 50x, and 100x target base coverage (*r* = 0.779, 0.790, and 0.792, respectively; p<0.01), as well as to other sequencing efficiency parameters (Table 2 and Fig 3A).

**Table 2:** Summary of correlation analyses between the pre-sequencing combined score and final sequencing parameters.

Parameter	Pearson correlation coefficient (*r*)	p-value
**Mean coverage**		
Overall dataset	0.628	< 0.01
20x coverage	0.779	< 0.01
50x coverage	0.790	< 0.01
100x coverage	0.792	< 0.01
**Target base coverage**		
*ATM* (rs659243)	0.650	< 0.01
*EYS* (rs9294631)	0.796	< 0.01
*KRAS* G12	0.703	< 0.01
*EGFR* L858	0.666	< 0.01
**Other parameters**		
Insert size	0.664	<0.01
Alignment rate	0.570	<0.01
Off-target rate	-0.423	<0.01
Base quality	0.476	<0.01

We next sought to define a pre-analytical cut-off value that could predict adequate sequencing results. For this end, we plotted the pre-sequencing variables (including the combined score) against the 50x coverage in our dataset, and defined 90% 50x coverage as a parameter for favorable results. According to this analysis, an FFPE storage time of 8.6 years, a PCR/QC ratio of 0.22, a DNA input of 960 ng, or a combined score of 266 were thresholds associated with sequencing efficiency (Fig 3B).

### Low depth of coverage was characteristic of regions with low GC content

**Fig 4:**
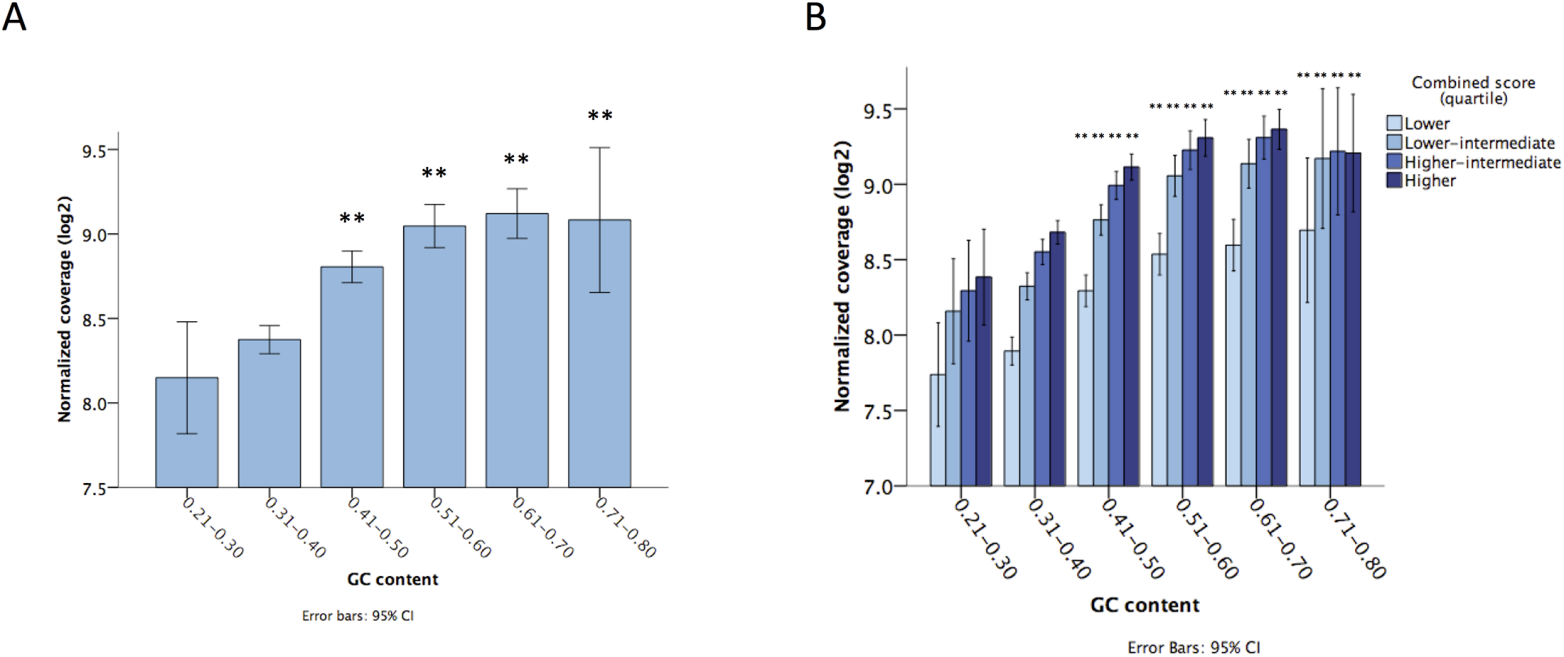
Normalized depth of coverage presents wide variability within the genome, with worse coverage observed in regions with lower GC content (A). The GC content effect was additive to sample quality to predict depth of coverage, as observed after stratifying sample quality using the combined score (B). Abbreviations: St. Dev., standard deviation. Obs: ** indicates significance at p<0.01.

In addition to sample quality, we evaluated the effect of base composition on sequencing depth. To assess depth of coverage uniformity, we evaluated the mean normalized coverage across the genomic regions spanned by the designed probes. We demonstrated a wide variability, and observed that poor coverage was significantly associated with regions presenting lower GC content (Fig 4A). The best coverage was observed in regions with 0.5–0.7 GC content ratios, with a marked deterioration below 0.4 (p<0.01). Subsequently, we stratified samples according to baseline quality (measured by the pre-sequencing combined score), and re-assessed the GC content effect. Notably, regions with low GC content (below 0.4) had worse coverage in every stratum, with a pronounced lower coverage in samples with poor pre-sequencing quality (Fig 4B).

### Impact of coverage variability on gene hotspots

As sequencing metrics are ultimately a surrogate for optimal variant calling, we interrogated if sample quality and GC content would have an impact on target coverage at hotspot positions in *KRAS* and *EGFR*. As shown in Table 3, these genes exemplify the opposite extremes of the coverage spectrum observed herein. While hotspot positions in *EGFR* presented an ideal GC content (0.51–0.55) and an optimal coverage, *KRAS* showed lower GC content (0.33–0.36) and a dramatically worse coverage. The median number of reads in the *KRAS* codon 12 was only 51 (range 3–183), and the 20x and 50x target coverage were 87.9% and 51.4%, respectively. On the other hand, all *EGFR* hotspot positions presented satisfactory coverage (Table 3). As NGS variant calling pipelines will often include filters bases on a minimal coverage (eg. 20x or 50x), recurrent *KRAS* mutations could be easily missed due to low coverage. In fact, 12 out of 22 *KRAS* mutant cases were detected among samples with 50 reads or less, and 3 cases were found with less than 20 reads (Table F in S1 File), all of which were confirmed by visual read inspection. Poor coverage in these sites could also impair the sensitivity to detect low allele frequency mutations. Using a minimum combined score threshold of 266, the median number of reads in the *KRAS* codon 12 was 72.5 (range 18–183), and the 20x and 50x coverage was 98.2% and 80.4%. In line with recent reports, we observed a negative correlation between the PCR/QC ratio and the dinucleotide CpG to TpG transitions (*r* = -0.186; p = 0.049). Similar correlations were not seen to other pre-analytical variables or other dinucleotide changes.

**Table 3:** GC content and depth of coverage at hotspot positions for recurrent somatic mutations in non-small cell lung cancer.

Genes	Codon	Exon	Chr position	GC content	Depth
					Median (range)
*KRAS*	G12	2	Chr12:25,398,285	0.33	51 (3–183)
	Q61	3	Chr12:25,380,276	0.36	741(161–1,513)
*PIK3CA*	E545	10	Chr3:178,936,091	0.30	159 (23–312)
	H1047	20	Chr3:178,952,085	0.37	1,067 (210–1864)
*BRAF*	V600	15	Chr7:140,453,136	0.34	459 (95–867)
*NRAS*	Q61	3	Chr1:115,256,529	0.40	648 (69–1,512)
*MEK1*	Q56	2	Chr15:66,727,451	0.50	808 (118–1,525)
	D67	2	Chr15:66,727,483	0.50	1,204 (188–2,185)
*EGFR*	G719	18	Chr7:55,241,708	0.51	1,409 (289–2,940)
	Dels	19	Chr7:55,242,470	0.53	503 (125–1,224)
	T790	20	Chr7:55,249,071	0.55	1,534 (344–3,019)
	L858	21	Chr7:55,259,524	0.54	918 (190–1,826)
*ERBB2*	G776	20	Chr17:37,880,996	0.58	930 (111–1593)

Abbreviations: Chr, chromosome; Dels, deletions.

## Discussion

In the present study, we confirmed that clinical FFPE samples are a reliable source of DNA for targeted gene sequencing in cancer, provided adequate sample quality controls are exercised. We demonstrated that three pre-analytical variables—FFPE storage time, PCR/QC ratio, and DNA input in the library preparation—were significantly correlated to most parameters of sequencing efficiency. The combined examination of these features may be particularly useful to define sample adequacy for sequencing, as demonstrated by a pooled model derived from them, which was highly correlated to sequencing efficiency. We also showed a significant variability in depth of coverage within the genome, dependent on the GC content ratio. Genomic regions with lower GC content presented worse depth of coverage, and this effect was additive to sample quality.

It has been shown that NGS data from FFPE samples have smaller library insert sizes and greater coverage variability.[23] Herein, we went further to demonstrate that FFPE storage time, PCR/QC ratio, and DNA input may all predict sequencing quality within this group. For instance, FFPE storage time (or tumor age) was negatively correlated to several post-sequencing parameters, including depth of coverage, insert size, and base quality. These results are in line with the findings from Hedegaard et al,[12] who also showed better results when more recently obtained FFPE samples were used. In this sense, several factors may have negatively affected the results in older samples, including non-standardized methods used in the past for tumor fixation, processing, embedding, as well as storage time *per se*. On the other hand, Schweiger et al[39] did not find an influence of tumor age on sequencing depth, however that study was limited by a very small sample size (only 7 FFPE samples). Although older tumors may be an exception in the clinical setting, pathologists may need to utilize ancient FFPE samples in specific research settings. Some possible scenarios include retrospective analysis of unique samples acquired in clinical trials, study of rare diseases, and when tissue bank samples are the only available source. If older samples are to be included, it may be essential to select for those with better DNA quality (using estimations of DNA fragmentation). When alternative sources are not an option, increasing the DNA input or the sequencing depth may help overcome the intrinsic limitations related to longer sample storage and older handling methods.

Different methods have been reported to assess quality of gDNA derived from clinical FFPE samples. These include verifying the A260/280 ratio using Nanodrop spectrophotometer (a ratio of 1.8 or greater suggests reasonable purity), calculating the estimated double-stranded DNA amount dividing Qubit^**®**^ DNA estimation by Nanodrop (ratios of 0.4 or higher are ideal), running an aliquot of gDNA on an agarose gel or TapeStation (fragments of 200 bp or less indicate poor quality), or using a PCR-based approach.[30–33] In this study, we utilized a standard protocol recommended by the manufacturer, based on the PCR amplification of genomic regions of different sizes.[35] Low quality DNA will generate less abundant amplicons, simulating the expected results during target enrichment. This kind of analysis was independently correlated to depth of coverage, with higher ratios giving the best coverage. This PCR-based test is relatively straightforward, inexpensive, uses low amount of DNA as input, and are therefore easily applicable in most laboratories.

DNA input in the library preparation is an important predictor of sequencing success.[21] For the platform used in the current study, the manufacturer recommends a minimum of 225 ng of gDNA (estimated by fluorescence methods like PicoGreen^**®**^ or Qubit^**®**^), which may be increased in the case of low quality DNA. Our data show that DNA input was correlated to sequencing depth, alignment rate, base quality, and off-target rate. More importantly, DNA input was interchangeable with other pre-sequencing parameters (tumor age, PCR/QC) to predict sequencing depth. It means that higher DNA input may often compensate for low-quality DNA, while high-quality DNA could be used at substantially lower input, as shown by the combined score analysis presented herein.

We generated a unique score that takes into account data from three pre-analytical variables that demonstrated independent impact on sequencing depth. Furthermore, we speculated on potential cut-off values for each of these variables that could help define tissue adequacy for sequencing. Though it may be attractive to consider these values in the routine of sequencing laboratories, some limitations need to be discussed. For instance, it is still uncertain if this evaluation could be applied to other settings, especially if distinct QC or NGS assays are employed. In addition, due to the retrospective nature of the current study, some specific data on histological processing could not be retrieved, like the amount of time spent between specimen collection and formalin fixation (ischemic time) and time spent in formalin fixation prior to processing. These and other non-assessable pre-analytical variables could have an additional impact on downstream sequencing analysis. Lastly, cut-off values may require local validation before implementation, and different metrics—like depth of coverage at specific hotspots—may be preferred as surrogates for optimal sequencing. Nonetheless, the concept of interchangeability between the pre-sequencing variables is likely universal, emphasizing the need to assess them in combination.

It has been demonstrated that hybridization-capture techniques may present significant coverage variability across the genome, with some nuances according to different platforms and base composition.[34, 40] In the current study, we confirmed a wide variability in depth of coverage within the target regions, with the best results achieved in genomic regions with 0.5–0.7 GC content ratios. On the other hand, genomic regions characterized by low GC content (below 0.4) presented significantly worse coverage, which was likely due to impaired probe hybridization and PCR amplification in these regions during library preparation.[34, 40] This effect was additive to sample quality in predicting sequencing depth. According to our analysis, regions with low GC content may be the most affected if samples with poor quality are used, achieving worse results in terms of depth of coverage. As different hybridization-capture platforms are currently available,[41] choosing those with the highest sequencing uniformity may be essential, especially when dealing with low-quality DNA.

In summary, we confirmed that FFPE samples are a reliable source for targeted gene sequencing in cancer, and validated sample storage time, DNA input, and DNA quality assessment as pre-analytical predictors of final sequencing metrics. We also showed that these variables are interchangeable (one variable could compensate for others), as demonstrated by a combined score analysis. Lastly, samples with poor quality yielded significantly worse sequencing results in genomic regions characterized by low GC content like in *KRAS*, emphasizing the relevance of sample quality controls.

## Supporting Information

S1 Fig The high rate of similar reads generated with Haloplex is exemplified by repetitive reads in a targeted exonic region in *EGFR*. (TIFF)

S2 Fig Representative images of the tumor cellularity among samples assessed with targeted gene sequencing. Tissue microarray (TMA) images were selected for convenience, although TMAs were not used in the DNA extraction process prior to sequencing.
(TIFF)

S3 Fig Median number of reads (A) and target base coverage (B) across 113 lung tumor samples assessed with targeted gene sequencing. Each point represents a unique case. Abbreviation: PF, passing filter; PCT, percentage.
(TIFF)

S1 File Supplemental tables A-F. (DOCX)
