# Supplementary figures and images for: Impact of Pre-Analytical Variables on Cancer Targeted Gene Sequencing Efficiency
Source: PLoS One. 2015 Nov 25;10(11):e0143092. doi: 10.1371/journal.pone.0143092 (PMC4659597; doi:10.1371/journal.pone.0143092)

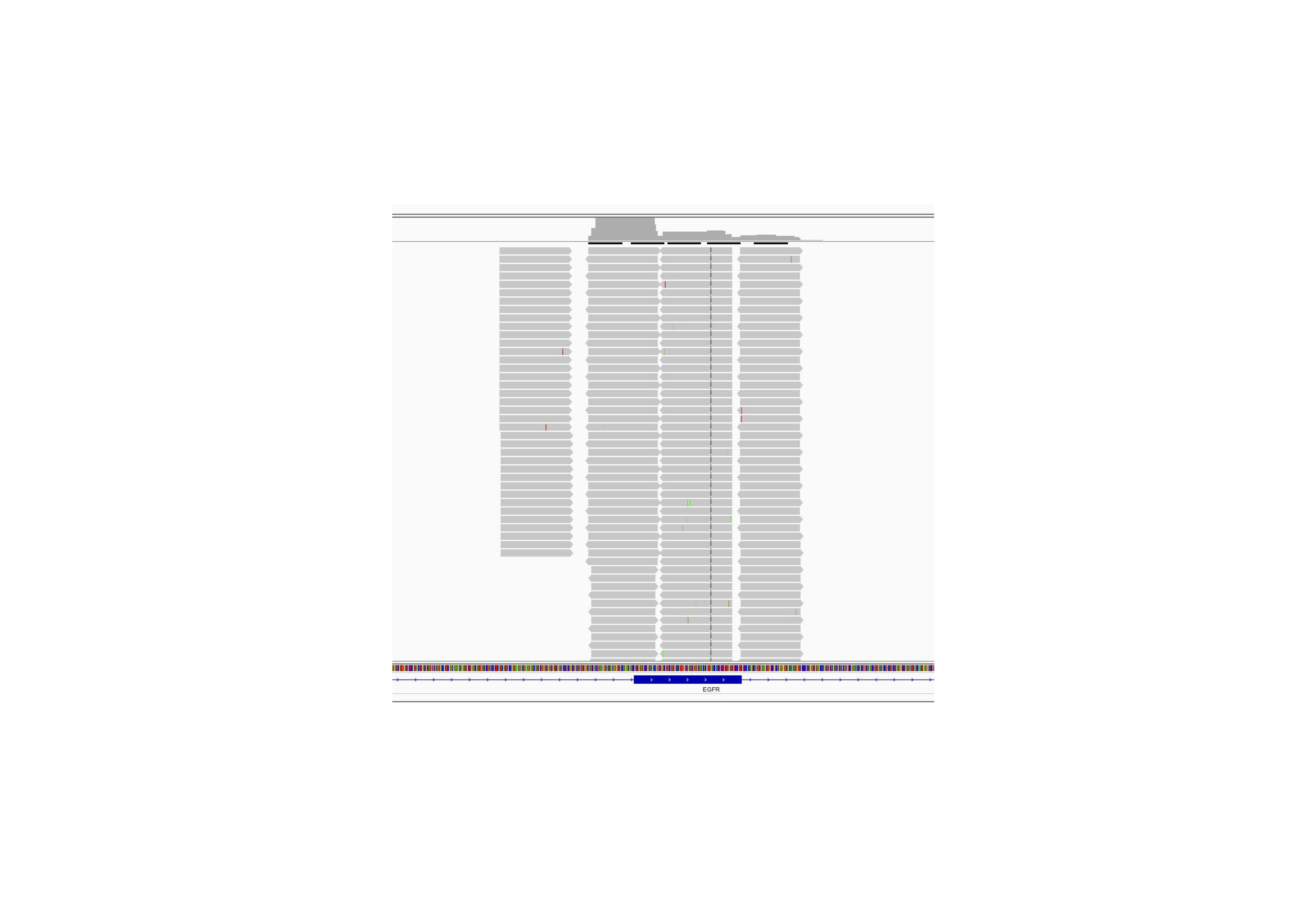

Supplement: S1 Fig — (TIFF) [file pone.0143092.s001.tiff]

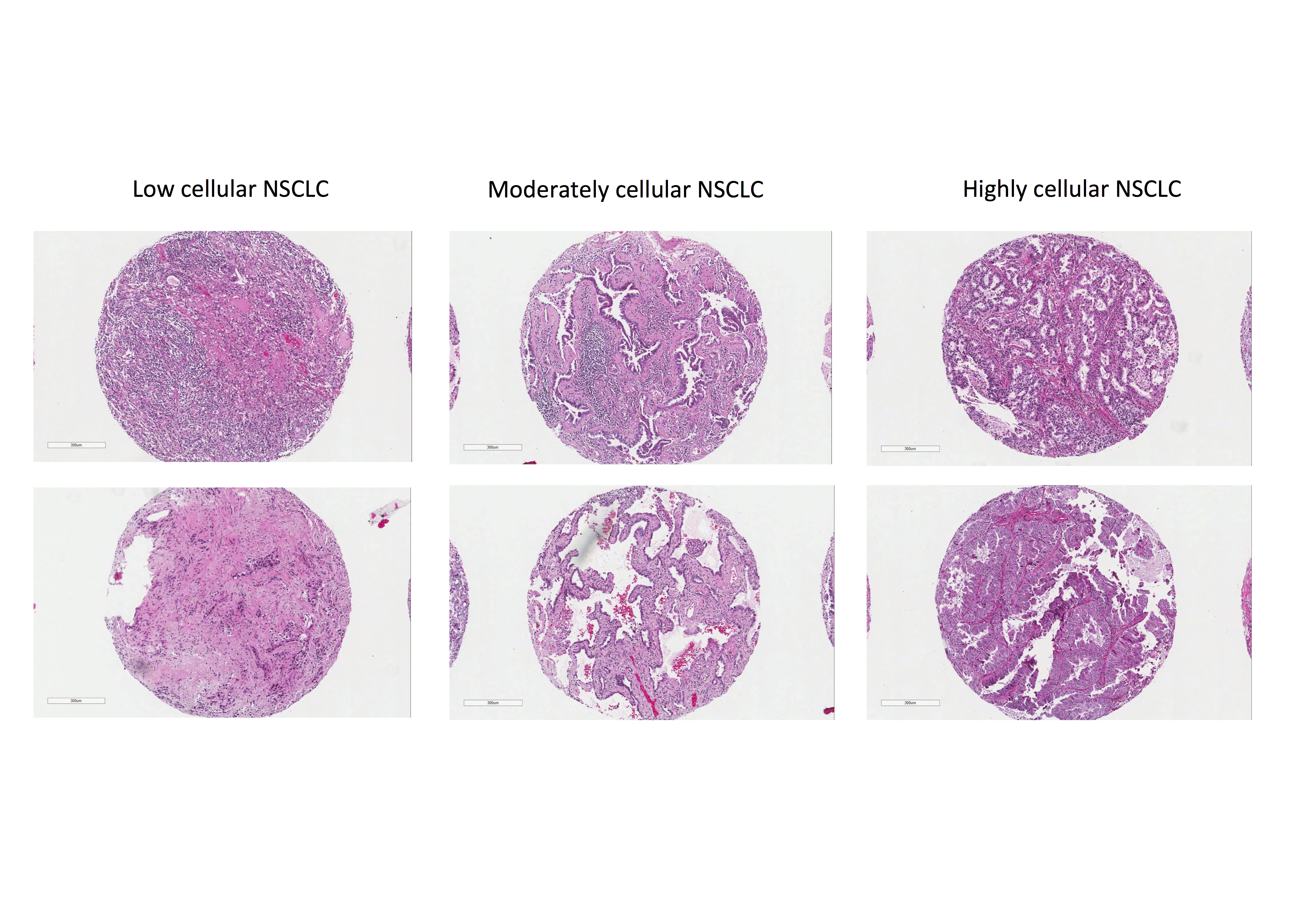

Supplement: S2 Fig — Tissue microarray (TMA) images were selected for convenience, although TMAs were not used in the DNA extraction process prior to sequencing. (TIFF) [file pone.0143092.s002.tiff]

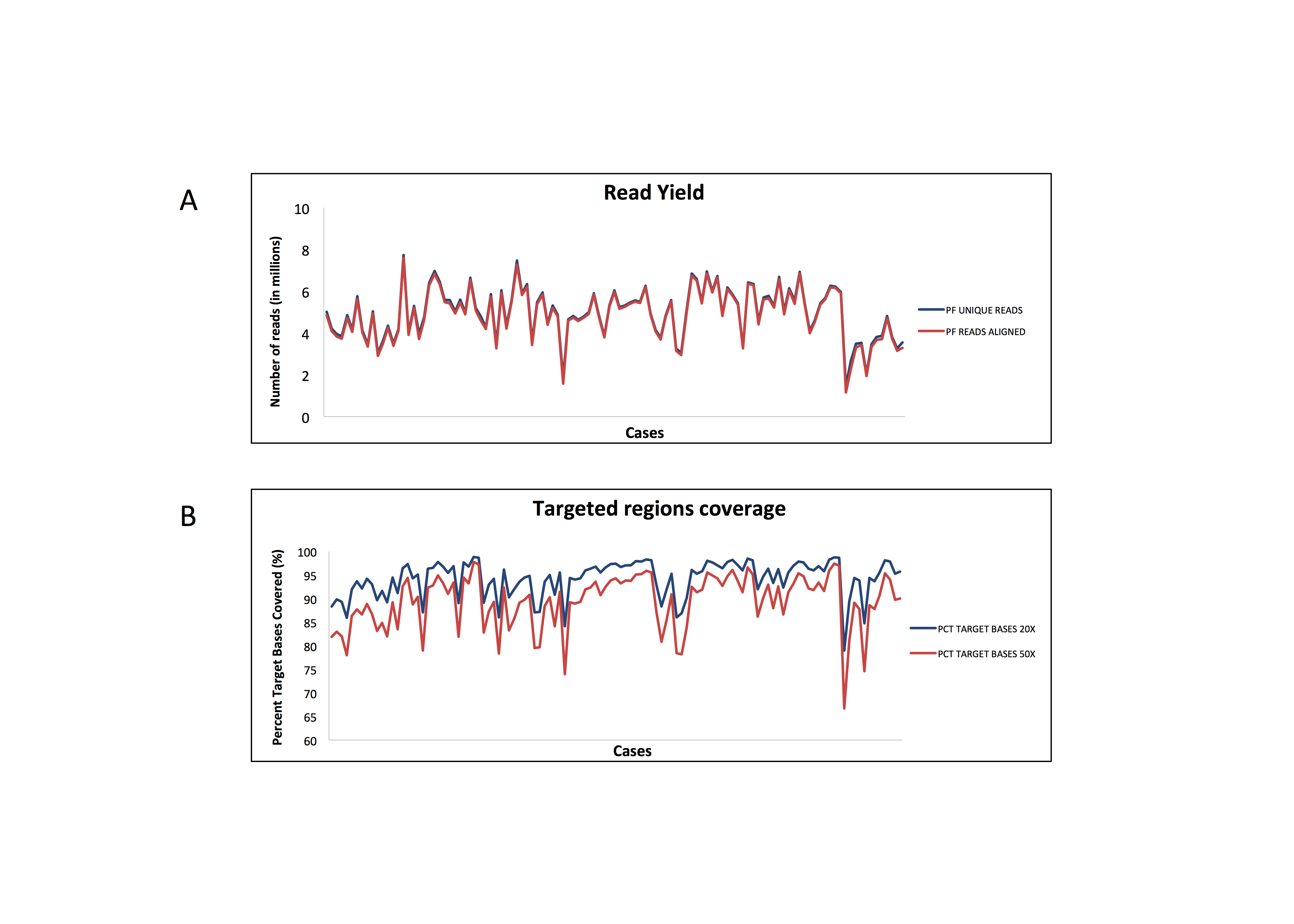

Supplement: S3 Fig — Each point represents a unique case. Abbreviation: PF, passing filter; PCT, percentage. (TIFF) [file pone.0143092.s003.tiff]
