# Supplementary material for: Impact of Pre-Analytical Variables on Cancer Targeted Gene Sequencing Efficiency
Source: PLoS One. 2015 Nov 25;10(11):e0143092. doi: 10.1371/journal.pone.0143092 (PMC4659597; doi:10.1371/journal.pone.0143092)
Supplement: S1 File — (DOCX) [file pone.0143092.s004.docx]

**S1 File**

Table A – Baseline characteristics (N=113).

| Characteristic | Frequency (%) |
| --- | --- |
| Primary tumor  Lung  Head and Neck | 110 (97.3%)  3 (2.7%) |
| Histology  Adenocarcinoma  Squamous  Adenosquamous  Others | 60 (53.1%)  34 (30.1%)  10 (8.8%)  9 (8.0%) |
| *EGFR* status  Mutant  Wild type | 7 (6.2%)  106 (93.8%) |
| *KRAS* status  Mutant  Wild type | 22 (19.5%)  91 (80.5%) |

Table B: Tumor cellularity of samples.

| **Sample** | **Cellularity** | **Sample** | **Cellularity** | **Sample** | **Cellularity** |
| --- | --- | --- | --- | --- | --- |
| OSU001 | 1 | OSU041 | 3 | OSU081 | 3 |
| OSU002 | 3 | OSU042 | 3 | OSU082 | 3 |
| OSU003 | 2 | OSU043 | 3 | OSU083 | 3 |
| OSU004 | 3 | OSU044 | 3 | OSU084 | 3 |
| OSU005 | 2 | OSU045 | 3 | OSU085 | 3 |
| OSU006 | 3 | OSU046 | 2 | OSU086 | 3 |
| OSU007 | 3 | OSU047 | 1 | OSU087 | 1 |
| OSU008 | 2 | OSU048 | 3 | OSU089 | 2 |
| OSU009 | 1 | OSU049 | 3 | OSU090 | 2 |
| OSU010 | 2 | OSU050 | 3 | OSU091 | 3 |
| OSU011 | 1 | OSU051 | 2 | OSU092 | 3 |
| OSU012 | 2 | OSU052 | 2 | OSU093 | 3 |
| OSU013 | 3 | OSU053 | 3 | OSU094 | 3 |
| OSU014 | 3 | OSU054 | 3 | OSU095 | 1 |
| OSU015 | 3 | OSU055 | 3 | OSU096 | 2 |
| OSU016 | 2 | OSU056 | 3 | OSU097 | 3 |
| OSU017 | 3 | OSU057 | 3 | OSU098 | 3 |
| OSU018 | 3 | OSU058 | 2 | OSU099 | 3 |
| OSU019 | 3 | OSU059 | 3 | OSU100 | 3 |
| OSU020 | 2 | OSU060 | 2 | OSU101 | 3 |
| OSU021 | 1 | OSU061 | 3 | OSU102 | 3 |
| OSU022 | 2 | OSU062 | 3 | K01 | 3 |
| OSU023 | 2 | OSU063 | 3 | K03 | 3 |
| OSU024 | 3 | OSU064 | 1 | K04 | 3 |
| OSU025 | 1 | OSU065 | 1 | K05 | 3 |
| OSU026 | 3 | OSU066 | 2 | K06 | 3 |
| OSU027 | 1 | OSU067 | 2 | K07 | 2 |
| OSU028 | 3 | OSU068 | 1 | K08 | 2 |
| OSU029 | 3 | OSU069 | 3 | K09 | 3 |
| OSU030 | 3 | OSU070 | 2 | K10 | 2 |
| OSU031 | 3 | OSU071 | 3 | K11 | 3 |
| OSU032 | 3 | OSU072 | 2 | K12 | 3 |
| OSU033 | 1 | OSU073 | 3 | K13 | 2 |
| OSU034 | 3 | OSU074 | 2 |  |  |
| OSU035 | 2 | OSU075 | 3 |  |  |
| OSU036 | 3 | OSU076 | 3 |  |  |
| OSU037 | 3 | OSU077 | 3 |  |  |
| OSU038 | 3 | OSU078 | 3 |  |  |
| OSU039 | 3 | OSU079 | 2 |  |  |
| OSU040 | 2 | OSU080 | 3 |  |  |

Obs: Tumor cellularity was determined by visual inspection of the number of tumor nuclei compared to stromal cell nuclei in the marked areas, and samples were classified as: low cellular (1), containing sparse tumor cells within fibrous or inflammatory background; moderately cellular (2); highly cellular (3), mostly tumor cells without significant intervening stroma, inflammation or airspaces. Most cases were estimated as either highly or moderately cellular (88%).

Table C: List of 81 genes selected based on relevance in non-small cell lung cancer.

| *AKT1* | *AXL* | *CREBBP* | *FAM123B* | *KIT* | *NF1* | *PPP2R1A* | *SMARCA4* | *WWOX* |
| --- | --- | --- | --- | --- | --- | --- | --- | --- |
| *AKT2* | *BCL11A* | *CRKL* | *FBLN2* | *KRAS* | *NFE2L2* | *PTEN* | *SOX2* |  |
| *AKT3* | *BCL2L1* | *CSMD1* | *FBXW7* | *LMTK2* | *NOTCH1* | *PTPRD* | *STK11* |  |
| *ALK* | *BRAF* | *CTNNB1* | *FGFR1* | *LRP1B* | *NOTCH2* | *RAF1* | *TNFAIP3* |  |
| *ANP32C* | *CBL* | *CUL3* | *FGFR2* | *MAP2K1* | *NOTCH3* | *RB1* | *TP53* |  |
| *APC* | *CCND1* | *EGFR* | *FGFR3* | *MCL1* | *NOTCH4* | *RBM10* | *TP63* |  |
| *ARAF* | *CDK4* | *EPHA3* | *FGFR4* | *MET* | *NRAS* | *REL* | *TSC1* |  |
| *ARID1A* | *CDK6* | *ERBB2* | *FOXP1* | *MLL2* | *PDGFRA* | *RET* | *U2AF1* |  |
| *ASCL4* | *CDKN2A* | *ERBB4* | *HRAS* | *MTOR* | *PDYN* | *ROS1* | *VGLL4* |  |
| *ATM* | *CDKN2B* | *EYS* | *KEAP1* | *MYC* | *PIK3CA* | *SMAD4* | *WHSC1L1* |  |

Table D: Correlation between pre-analytical and post-analytical parameters.

| Variable | Parameters | Storage time | Off-target | PCR/QC | Insert size | DNA input | Total reads | Target reads | Align rate | Base quality |
| --- | --- | --- | --- | --- | --- | --- | --- | --- | --- | --- |
| Storage time | Pearson  p-value | 1 | .285  .002 | -.338  .000 | -.764  .000 | -.116  .222 | -.356  .000 | -.405  .000 | -.354  .000 | -.188  .046 |
| Off-target | Pearson  p-value | .285  .002 | 1 | -.086  .378 | -.216  .021 | -.336  .000 | -.575  .000 | -.589  .000 | -.617  .000 | -.334  .000 |
| PCR/QC | Pearson  p-value | -.338^*^  .000 | -.086  .378 | 1 | .601  .000 | -.224  .020 | .131  .178 | .183  .058 | .169  .080 | .162  .094 |
| Insert size | Pearson  p-value | -.764  .000 | -.216  .021 | .601  .000 | 1 | .081  .395 | .363  .000 | .423  .000 | .307  .001 | .258  .006 |
| DNA input | Pearson  p-value | -.116  .222 | -.336  .000 | -.224  .020 | .081  .395 | 1 | .548  .000 | .549  .000 | .449  .000 | 477  .000 |
| Total reads | Pearson  p-value | -.356  .000 | -.575  .000 | .131  .178 | .363  .000 | .548  .000 | 1 | .996  .000 | .624  .000 | .471  .000 |
| Target reads | Pearson  p-value | -.405  .000 | -.589  .000 | .183  .058 | .423  .000 | .549  .000 | .996  .000 | 1 | .650  .000 | .485  .000 |
| Align rate | Pearson | -.354  .000 | -.617  .000 | .169  .080 | .307  .001 | .449  .000 | .624  .000 | .650  .000 | 1 | .742  .000 |
| Base quality | Pearson  p-value | -.188  .046 | -.334  .000 | .162  .094 | .258  .006 | .477^**^  .000 | .471  .000 | .485  .000 | .742  .000 | 1 |

Abbreviations: Pearson, Pearson correlation coefficient; PCR/QC, PCR-based quality control; Align rate, alignment rate.

Table E: Multivariate analysis evaluating the effect of pre-sequencing parameters on depth of coverage.

|  | Estimate | Standard error | p-value |
| --- | --- | --- | --- |
| (Intercept) | 202.95 | 26.91 | <0.01 |
| Tumor age | -7.86 | 1.31 | <0.01 |
| PCR/QC ratio | 249.95 | 55.04 | <0.01 |
| DNA input | 0.08 | 0.01 | <0.01 |

Abbreviations: PCR/QC, PCR-based quality control.

Table F: Summary of sequencing metrics in *KRAS* mutant samples.

| Sample | Quality | Depth | VAF | Chr | Start | End | Ref | Alt | Gene | Protein |
| --- | --- | --- | --- | --- | --- | --- | --- | --- | --- | --- |
| K003 | 120.7 | 17 | 0.58 | 12 | 25398284 | 25398284 | C | T | *KRAS* | G12D |
| K004 | 109.7 | 36 | 0.16 | 12 | 25398285 | 25398285 | C | A | *KRAS* | G12C |
| K008 | 255.7 | 58 | 0.25 | 12 | 25398285 | 25398285 | C | A | *KRAS* | G12C |
| K009 | 319.7 | 81 | 0.25 | 12 | 25398284 | 25398284 | C | A | *KRAS* | G12V |
| K012 | 130.7 | 26 | 0.26 | 12 | 25398284 | 25398284 | C | A | *KRAS* | G12V |
| K013 | 57.7 | 47 | 0.12 | 12 | 25398284 | 25398284 | C | A | *KRAS* | G12V |
| AA004 | 40.77 | 6 | 0.33 | 12 | 25398285 | 25398285 | C | A | *KRAS* | G12C |
| AA006 | 485.77 | 33 | 0.60 | 12 | 25398285 | 25398285 | C | A | *KRAS* | G12C |
| AA013 | 12.99 | 44 | 0.11 | 12 | 25398285 | 25398285 | C | A | *KRAS* | G12C |
| AA018 | 64.77 | 31 | 0.16 | 12 | 25398285 | 25398285 | C | A | *KRAS* | G12C |
| AA030 | 384.77 | 160 | 0.14 | 12 | 25398285 | 25398285 | C | T | *KRAS* | G12S |
| AA037 | 30.77 | 16 | 0.25 | 12 | 25398284 | 25398284 | C | G | *KRAS* | G12A |
| AA038 | 49.77 | 48 | 0.16 | 12 | 25398284 | 25398284 | C | A | *KRAS* | G12V |
| AA043 | 267.77 | 24 | 0.5 | 12 | 25398284 | 25398284 | C | A | *KRAS* | G12V |
| AA044 | 745.77 | 69 | 0.43 | 12 | 25398284 | 25398284 | C | A | *KRAS* | G12V |
| AA063 | 282.77 | 107 | 0.14 | 12 | 25398285 | 25398285 | C | A | *KRAS* | G12C |
| AA068 | 19.81 | 51 | 0.09 | 12 | 25398284 | 25398284 | C | G | *KRAS* | G12A |
| AA070 | 57.77 | 39 | 0.10 | 12 | 25398284 | 25398284 | C | A | *KRAS* | G12V |
| AA073 | 172.77 | 51 | 0.192 | 12 | 25398284 | 25398284 | C | T | *KRAS* | G12D |
| AA077 | 614.77 | 72 | 0.43 | 12 | 25398284 | 25398284 | C | G | *KRAS* | G12A |
| AA080 | 678.77 | 120 | 0.24 | 12 | 25398285 | 25398285 | C | A | *KRAS* | G12C |
| AA082 | 1880.77 | 105 | 0.65 | 12 | 25398282 | 25398282 | C | A | *KRAS* | G13C |

Abbreviations: Chr, chromosome; Del-Infr, inframe deletion; VAF, variant allele frequency.
